# Pre-operative soft tissue injuries as independent predictors of elbow stiffness after radial head fracture fixation: a pilot study

**DOI:** 10.1016/j.jseint.2026.101652

**Published:** 2026-01-29

**Authors:** Ji-Ho Lee, Gwang-Sub Lee, Christopher W. Jenkins, Kee-Baek Ahn, Byoungjoo Lee, In Hyeok Rhyou

**Affiliations:** a Department of Orthopaedic Surgery, Pohang SM Christianity Hospital, Pohang, Republic of Korea; b Department of Orthopaedic Surgery, Manchester University NHS Foundation Trust, Manchester, UK; c Shiley Center for Orthopaedic Research and Education at Scripps Clinic, La Jolla, CA, USA

**Keywords:** Radial head fractures, Elbow joint, Post-operative stiffness, Soft tissue injuries, Pilot study, Immobilization

## Abstract

**Background:**

Post-operative elbow stiffness is a common and debilitating complication following open reduction and internal fixation for displaced radial head fractures. The relative contributions of post-operative immobilization and intrinsic soft tissue damage remain debated. The purpose of this pilot study was to generate preliminary data on this relationship to inform the design of future, definitive research.

**Methods:**

This study retrospectively reviewed 45 patients who underwent open reduction and internal fixation for displaced radial head fractures. Pre-operative magnetic resonance imaging was used to identify injuries to the anterior capsule (AC), posterior band of the medial collateral ligament (pMCL), and lateral collateral ligament complex. Primary outcomes were final range of motion. For descriptive purposes, patients were categorized by immobilization duration (<3 weeks vs. ≥3 weeks). To assess independent predictors of final motion, multivariate linear regression analysis was performed, treating immobilization duration as a continuous variable (in days).

**Results:**

No statistically significant differences in final range of motion were found between the short and long immobilization groups. Specific soft tissue injuries were strongly associated with motion loss. AC tears were linked to greater extension loss (mean deficit 15.0° vs. 2.0°, *P* < .001), and pMCL tears were associated with reduced flexion (mean 132.8° vs. 142.7°, *P* < .001). In the multivariate analysis, an AC tear was the only significant independent predictor of final extension loss (*P* < .001), and a pMCL tear was the only significant predictor of final flexion (*P* < .001). Immobilization duration, when analyzed as a continuous variable, was not a significant predictor of either flexion or extension loss.

**Conclusion:**

This pilot study provides strong preliminary evidence that specific soft tissue injuries, particularly of the AC and pMCL, are independent predictors of post-operative stiffness, whereas immobilization duration is not. The study was underpowered to definitively rule out a small effect of immobilization. These findings justify the need for a large, multicenter trial to confirm these associations and establish evidence-based post-operative guidelines.

Radial head fractures are among the most common elbow injuries, accounting for approximately one-third of all elbow fractures.^2^^,^^3^ These injuries frequently result from a fall on an outstretched hand, leading to axial and valgus forces that disrupt the radiocapitellar joint.^16^ While nondisplaced fractures are often managed nonoperatively with favorable results,^6^ displaced or comminuted fractures—especially those associated with mechanical blocks or instability—typically require surgical intervention.^19^^,^^23^ Open reduction and internal fixation (ORIF) is often the preferred treatment for reconstructible fractures, aiming to restore anatomic alignment and preserve the stabilizing function of the radial head.^23^^,^^24^ However, a frequent and debilitating complication following ORIF is post-operative elbow stiffness, which can significantly impair functional recovery and quality of life.^15^

For decades, it has been widely accepted in clinical practice that extended post-operative immobilization is one of the main causes of elbow contracture.^13^^,^^15^^,^^25^ This concern has led to a widespread emphasis on initiating early range of motion (ROM) to mitigate the risk of stiffness, a principle supported by observations in other elbow injuries, such as dislocations, where immobilization beyond four weeks can lead to severe contractures.^14^ Despite this guiding principle, the optimal duration of immobilization after stable ORIF for isolated radial head fractures remains poorly defined. Post-operative protocols differ among surgeons, with some recommending early motion and others suggesting several weeks of protection.^10^ This variation reflects a lack of consensus in the available evidence for managing these injuries.

Recent research indicates that intrinsic tissue damage incurred during the initial injury may serve as a more significant biological determinant of stiffness than the extrinsic factor of immobilization.^4^ Radial head fractures are frequently accompanied by significant injuries to the surrounding soft tissues, including the joint capsule and collateral ligaments.^17^^,^^20^^,^^21^ Such injuries can initiate a cascade of inflammation and subsequent fibrosis, leading to the development of a stiff, noncompliant periarticular environment that resists motion despite appropriate rehabilitation.^5^^,^^7^ While the general association between soft tissue trauma and elbow stiffness is recognized, the specific contributions of individual capsuloligamentous structures to distinct patterns of motion loss have not been well elucidated in this patient population.

Therefore, the purpose of this pilot study was to conduct an initial investigation into the relative contributions of immobilization duration and specific, magnetic resonance imaging (MRI)–documented soft tissue injuries to post-operative stiffness following ORIF of displaced radial head fractures. The study was designed to address 2 primary questions reflective of its preliminary nature: (1) to gather preliminary data comparing functional outcomes between patients with short (<3 weeks) vs. long (≥3 weeks) immobilization periods and to perform a power analysis to determine the required sample size for a definitive study; and (2) to identify which specific soft tissue injuries demonstrate the strongest preliminary association with post-operative motion deficits. By exploring these factors, this study aims to generate a robust hypothesis and provide the foundational data necessary for designing a larger, prospective trial to establish evidence-based rehabilitation protocols.

## Materials and methods

### Study design and patient selection

This retrospective cohort study was conducted at a single orthopedic specialty institute following approval from the institutional review board (IRB No: PSMCHIRB-2023-002). The institutional database was queried for all patients treated for isolated radial head fractures between July 2010 and September 2020. During this period, 1,376 such patients were identified. Of these, 1,296 patients were treated conservatively, and 20 underwent radial head replacement. Consequently, only 60 patients (4.4%) were treated with ORIF. Inclusion criteria for this study were: (1) a displaced isolated radial head fracture treated with ORIF; (2) skeletally mature with closed elbow epiphyses; and (3) a minimum follow-up of 1 year. Patients were excluded if they had associated coronoid or capitellar fractures (excluding Osborne-Cotterill lesions),^8^ an associated triceps tendon rupture with a fleck sign,^11^ an ipsilateral forearm or humerus fracture, or clear evidence of elbow dislocation on imaging. Application of these criteria resulted in a final cohort of 45 patients for analysis.

### Surgical technique and post-operative management

All surgical procedures were performed by one of 3 fellowship-trained elbow surgeons. A lateral approach between the extensor carpi ulnaris and anconeus muscles was utilized in all cases.^9^ Fracture fixation was achieved using either radial head-specific locking plates (n = 17) or headless compression screws (n = 28), with the choice of implant at the discretion of the treating surgeon. After fixation of the radial head, associated ligamentous injuries were addressed. All identified tears of the lateral collateral ligament complex (LCLC) were repaired and reattached to their anatomic origin using suture anchors. If a manual valgus stress test performed at 30° of elbow flexion revealed instability, the anterior band of the medial collateral ligament (aMCL) was repaired via a separate medial approach. The posterior band of the medial collateral ligament (pMCL) was not repaired in any case.

Post-operatively, the duration of immobilization in a splint was determined by the treating surgeon based on intraoperative assessment of fracture stability and soft tissue integrity. For descriptive analysis, patients were dichotomized into 2 groups: a short immobilization group (group A, <3 weeks, n = 24) and a long immobilization group (group B, ≥3 weeks, n = 21). This 3-week cutoff was chosen as it represents a common clinical transition point from a protective phase to a more active rehabilitation phase.^22^ Following the immobilization period, all patients underwent a standardized rehabilitation protocol.

### Data collection and outcome measures

At the final follow-up visit (typically 6-12 months post-operatively), elbow ROM was measured by one of the authors using a standard goniometer. Flexion and extension were measured with the arm at the side, and forearm rotation (supination and pronation) was measured with the elbow flexed to 90°. Clinical outcomes were assessed using 2 validated instruments: the clinician-rated Mayo Elbow Performance Score, scored from 0 to 100 (higher scores indicate better function), and the patient-reported quick Disabilities of the Arm, Shoulder, and Hand questionnaire, scored from 0 to 100 (higher scores indicate greater disability).

### Magnetic resonance imaging protocol and interpretation

All patients underwent pre-operative MRI of the injured elbow within seven days of injury using 1.5-T scanners (Intera, Philips, Amsterdam, The Netherlands; or Signa HDx, GE Health care, Milwaukee, WI, USA). The imaging protocol included T1-weighted, T2-weighted, and T2 fat-saturated sequences in the coronal, sagittal, and axial planes. Three fellowship-trained upper limb orthopedic surgeons, blinded to patient information, independently reviewed all MRI scans to assess the integrity of the anterior capsule (AC), aMCL, pMCL, and LCLC.

**Figure 1:**
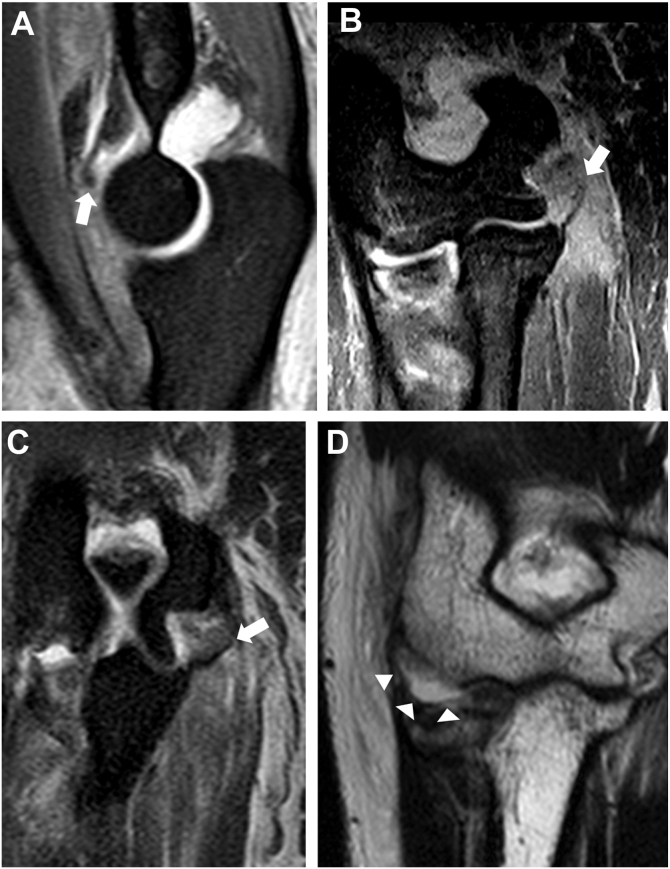
MRI findings of soft-tissue injuries. (**A**) Avulsion injury of the anterior capsule from the coronoid process. (**B**) Anterior medial collateral ligament tear from the medial epicondyle. (**C**) Detachment of the posterior medial collateral ligament from the posterior aspect of the medial epicondyle. (**D**) Lateral collateral ligament complex injury with distraction from the humeral origin. *MRI*, magnetic resonance imaging

A tear of the AC was defined as an avulsion from the coronoid process or a mid-substance disruption.^12^ An aMCL tear was defined as detachment from the medial epicondyle, a mid-substance tear, or detachment from the sublime tubercle.^1^ A pMCL tear was defined as detachment from the medial epicondyle or a mid-substance tear.^1^ An LCLC tear was defined as detachment from its humeral origin.^1^ (Fig. 1)

The reviewers reanalyzed the scans two weeks after their initial assessment to determine intraobserver reliability. Disagreements were resolved by consensus discussion.

### Statistical analysis

Statistical analyses were performed using Python Version 3.9.0 (Python Software Foundation, Wilmington, DE, USA). For descriptive purposes, continuous variables between the dichotomized immobilization groups were compared using independent *t*-tests, while categorical variables were compared using the chi-square or Fisher exact test. A Bonferroni correction was applied to adjust for multiple comparisons in the analysis of ROM and clinical scores, with a corrected significance level set at *P* < .05/6 ≈ 0.0083. A post hoc power analysis was conducted for this group comparison.

To avoid information loss caused by artificial dichotomization, 2 multivariate linear regression models were constructed to assess independent predictors of post-operative stiffness, using final flexion and final extension deficit as primary continuous outcomes. Independent variables included patient age, fixation type (plate vs. screw), the presence of AC, pMCL, and LCLC tears, and the duration of immobilization, treated as a continuous variable (in days).

Multicollinearity between predictors was assessed using the variance inflation factor, which quantifies the degree to which a predictor is correlated with other predictors in the model; a variance inflation factor below 5.0 was considered acceptable. All statistical tests were two-sided, and a *P* value <.05 was considered statistically significant.

## Results

### Patient demographics and injury characteristics

The final cohort consisted of 45 patients (24 males, 21 females) with a mean age of 44.8 years (range, 18-75 years). The 2 descriptively categorized immobilization cohorts were comparable in terms of mean age (group A: 44 years; group B: 40 years; *P* = .366) and fracture type distribution (*P* = .342). The cohort included 30 Mason type II and 15 Mason type III fractures. Fracture type did not significantly affect final flexion (*P* = .122), extension (*P* = .429), Mayo Elbow Performance Score (*P* > .2), or quick Disabilities of the Arm, Shoulder, and Hand scores (*P* > .2).

Inter- and intraobserver reliability for MRI interpretation of capsuloligamentous injuries was substantial (Kappa = 0.73, *P* < .001 and Kappa = 0.78, *P* < .001, respectively). MRI identified an anterior capsular tear in 23 patients (51.1%), a pMCL tear in 23 patients (51.1%), and an LCLC tear in 24 patients (53.3%). An aMCL tear was identified in 21 patients (46.7%); notably, all 21 patients with an aMCL tear also had a concurrent pMCL tear.

### Influence of immobilization duration

**Figure 2:**
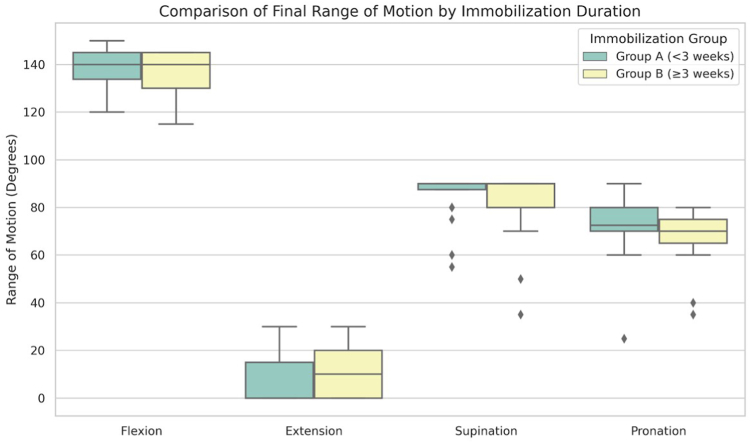
Box plot comparing final range of motion (flexion, extension, supination, and pronation) by post-operative immobilization duration. Patients were categorized into short (<3 weeks) and long (≥3 weeks) immobilization groups. The boxes represent the IQR, the horizontal line represents the median, and the whiskers extend to the most extreme data points within 1.5 times the IQR from the box edges. Outliers are shown as individual points. *IQR*, interquartile range.

The mean duration of immobilization was 11.0 ± 4.2 days in the short immobilization group (group A) and 28.9 ± 4.8 days in the long immobilization group (group B). In the descriptive comparison, there were no statistically significant differences in any of the primary outcome measures between these 2 groups at final follow-up (all *P* > .05) (Table I) (Fig. 2)

**Table I:** Comparison of clinical outcomes by immobilization duration.

Outcome measure	Group A	Group B	*P* value
	Immobilization period	Immobilization period	
	<3 wk	≥3 wk	
Flexion	138.8 ± 8.9	136.4 ± 8.8	.385
Extension	7.3 ± 9.9	10.2 ± 9.8	.322
Supination	85.4 ± 9.7	82.1 ± 14.9	.381
Pronation	71.9 ± 12.5	68.3 ± 11.4	.329
MEPS	92.3 ± 11.2	95.5 ± 8.5	.303
Q-DASH	4.8 ± 11.2	2.5 ± 5.5	.410

*MEPS*, Mayo Elbow Performance Score; *Q-DASH*, quick Disabilities of the Arm, Shoulder, and Hand.

Values are mean ± standard deviation.

### Power analysis for immobilization comparison

A post hoc power analysis was performed to evaluate the study's ability to detect the observed differences between the short and long immobilization groups. To detect the observed mean difference in final flexion (2.4°) with 80% power at an alpha level of 0.05, a total sample size of 233 patients would be required. Similarly, to detect the observed mean difference in extension loss (2.9°), a sample size of 175 patients would be needed. This analysis confirms that the present study, with a cohort of 45 patients, was substantially underpowered to detect small but potentially clinically meaningful differences in ROM between the immobilization groups.

### Association of soft tissue injuries with clinical outcomes

In contrast to the findings regarding immobilization, the occurrence of specific soft tissue injuries was statistically significantly associated with post-operative motion deficits (Table II). Patients with an AC tear had significantly worse elbow extension, with a mean extension deficit of 15.0° ± 9.3° compared to only 2.0° ± 4.8° in those with an intact capsule (*P* < .001). Flexion was also significantly reduced in the AC tear group (133.7° ± 9.5° vs. 141.8° ± 5.7°; *P* = .0013). Both of these findings remained highly significant after Bonferroni correction (*P* < .0083).

**Table II:** Post-operative range of motion and clinical outcome comparison based on the presence or absence of key soft tissue injuries (AC tear, pMCL tear, and LCLC tear).

Outcome measure	With AC tear	No AC tear	*P* value (AC)
Flexion (°)	133.7 ± 9.5	141.8 ± 5.7	**.0013**
Extension (°)	15.0 ± 9.3	2.0 ± 4.8	**<.001**
Supination (°)	81.7 ± 13.9	86.1 ± 10.2	.236
Pronation (°)	68.0 ± 11.7	72.5 ± 12.1	.217
MEPS	94.0 ± 11.6	93.8 ± 8.2	.952
Q-DASH	5.79 ± 11.7	1.6 ± 3.4	.123

Outcome measure	With pMCL tear	No pMCL tear	*P* value (pMCL)
Flexion (°)	132.8 ± 9.1	142.7 ± 4.8	**<.001**
Extension (°)	13.5 ± 9.9	3.6 ± 6.9	**<.001**
Supination (°)	80.7 ± 15.0	87.3 ± 7.7	.071
Pronation (°)	66.1 ± 14.5	74.5 ± 6.5	.016
MEPS	91.1 ± 12.1	96.4 ± 6.9	.087
Q-DASH	5.0 ± 10.2	2.4 ± 7.1	.348

Outcome measure	With LCLC tear	No LCLC tear	*P* value (LCLC)
Flexion (°)	136.7 ± 7.6	138.8 ± 10.1	.423
Extension (°)	10.0 ± 8.3	7.1 ± 11.4	.337
Supination (°)	82.9 ± 15.1	85.0 ± 8.4	.578
Pronation (°)	66.8 ± 14.3	74.0 ± 7.4	.044
MEPS	96.0 ± 8.7	91.8 ± 10.8	.178
Q-DASH	2.6 ± 6.2	4.8 ± 10.7	.430

*AC*, anterior capsule; *pMCL*, posterior band of medial collateral ligament; *LCLC*, lateral collateral ligament complex; *MEPS*, Mayo Elbow Performance Score; *Q-DASH*, quick Disabilities of the Arm, Shoulder, and Hand.

Values are mean ± SD. *P* values are from unpaired *t*-tests.

Bold indicates significance after Bonferroni correction for multiple comparisons (*P* < .0083).

Similarly, the presence of a pMCL tear was strongly associated with loss of motion. Patients with a pMCL tear had significantly reduced flexion (132.8° ± 9.1° vs. 142.7° ± 4.8°; *P* < .001) and a greater extension deficit (13.5° ± 9.9° vs. 3.6° ± 6.9°; *P* < .001). These differences also remained significant after Bonferroni correction. Because all aMCL tears occurred in conjunction with pMCL tears, it was not possible to statistically isolate the effect of an aMCL injury. The presence of an LCLC tear, which was surgically repaired in all cases, did not have a significant effect on final flexion or extension.

### Multivariate analysis for predictors of stiffness

**Figure 3:**
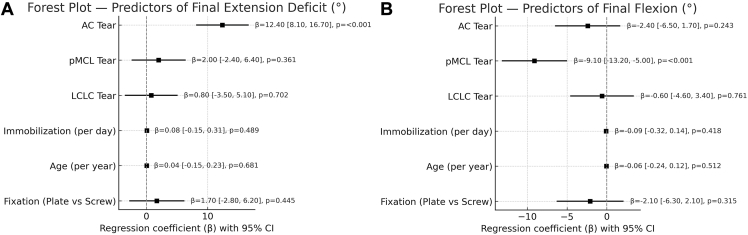
Forest plot of multivariate linear regression analysis for predictors of final range of motion. The points represent the β, and the horizontal lines represent the 95% CI. The vertical dashed line at zero indicates no effect. Predictors whose CIs do not cross the zero line are statistically significant. (**A**) Predictors for final extension deficit. (**B**) Predictors for final flexion. *CI*, confidence interval; *AC*, anterior capsule; *pMCL*, posterior band of the medial collateral ligament; *LCLC*, lateral collateral ligament complex; *β*, regression coefficient.

The results of the multivariate linear regression analysis are presented in Table III and Fig. 3. In the model predicting final extension deficit, the presence of an AC tear was the only significant independent predictor (β = 12.4°; *P* < .001). This indicates that, after adjusting for other factors, an AC tear was associated with an average increase of 12.4° in extension loss. The overall model explained 61% of the variance in final extension deficit (R2 = 0.61).

**Table III:** Multivariate linear regression analysis for predictors of final range of motion.

Outcome: Final extension deficit (°)				
Variable	Coefficient (β)	95% CI	*P* value	Clinical interpretation
AC tear	12.4	8.1-16.7	<.001	An AC tear was the strongest predictor, associated with an average of 12.4° more extension loss.
pMCL tear	2	−2.4 to 6.4	.361	Not a significant predictor
LCLC tear	0.8	−3.5 to 5.1	.702	Not a significant predictor
Immobilization (per d)	0.08	−0.15 to 0.31	.489	Not a significant predictor
Age (per yr)	0.04	−0.15 to 0.23	.681	Not a significant predictor.
Fixation (plate)	1.7	−2.8 to 6.2	.445	Not a significant predictor.

Outcome: final flexion (°)				
Variable	Coefficient (β)	95% CI	*P* value	Clinical interpretation
AC tear	−2.4	−6.5 to 1.7	.243	Not a significant predictor.
pMCL tear	−9.1	−13.2 to −5.0	<.001	A pMCL tear was the strongest predictor, associated with an average of 9.1° less final flexion.
LCLC tear	−0.6	−4.6 to 3.4	.761	Not a significant predictor
Immobilization (per d)	−0.09	−0.32 to 0.14	.418	Not a significant predictor.
Age (per yr)	−0.06	−0.24 to 0.12	.512	Not a significant predictor
Fixation (plate)	−2.1	−6.3 to 2.1	.315	Not a significant predictor

*AC*, anterior capsule; *pMCL*, posterior band of medial collateral ligament; *LCLC*, lateral collateral ligament complex; *CI*, confidence interval.

In the model for final flexion, the presence of a pMCL tear was the only significant independent predictor (β = −9.1°; *P* < .001). This suggests that a pMCL tear was associated with an average decrease of 9.1° in final flexion, independent of other factors. This model explained 48% of the variance in final flexion (R2 = 0.48). Multivariate linear regression was also performed to identify predictors for final pronation and supination. Unlike the flexion–extension arc, no specific soft tissue injury (AC, pMCL, or LCLC) was identified as a significant independent predictor for persistent deficits in supination or pronation (*P* > .05 for all variables). Notably, immobilization duration, when treated as a continuous variable, was not a significant predictor in either model (all *P* > .05).

## Discussion

This pilot study investigated the relative contributions of post-operative immobilization and specific soft tissue injuries to elbow stiffness following ORIF for displaced radial head fractures. The main result indicates that certain capsuloligamentous injuries observed on pre-operative MRI, specifically tears of the AC and pMCL, are independent indicators associated with particular patterns of motion loss following surgery. The strength of this association persisted following adjustment for potential confounding variables, such as immobilization duration, in a multivariate linear regression analysis. In contrast, immobilization duration, whether analyzed as a dichotomous or continuous variable, was not significantly associated with final ROM.

The data strongly suggest a dominant role for the AC and pMCL in the pathogenesis of post-traumatic elbow stiffness. The association between AC tears and a subsequent loss of extension is biomechanically intuitive. The AC is a primary static restraint to elbow extension, and injury to this structure can lead to inflammation, thickening, and fibrotic remodeling that mechanically blocks terminal extension.^4^^,^^7^^,^^25^ Our multivariate model confirmed that an AC tear was the most powerful independent predictor of extension loss, a finding consistent with animal models that have demonstrated a direct causal link between anterior capsular injury and the development of flexion contractures.^4^^,^^5^^,^^25^ Similarly, the strong link between pMCL tears and diminished final flexion is consistent with the ligament's biomechanics, as the posterior bundle becomes increasingly taut during deep elbow flexion.^16^^,^^18^ Injury and subsequent scarring of this structure can therefore create a fibrotic tether that restricts the joint's ability to achieve full flexion.^4^^,^^7^ This is supported by surgical studies demonstrating that release of the pMCL can be an effective technique for improving flexion in patients with established post-traumatic elbow stiffness.^18^

Regarding forearm rotation, our multivariate analysis did not identify any specific soft tissue injury (AC, pMCL, or LCLC) as an independent predictor for pronation or supination deficits. This stands in contrast to the clear associations found with the flexion–extension arc. We hypothesize several reasons for this finding. First, forearm rotation is a complex motion involving not only the radiocapitellar joint but also the distal radioulnar joint and the interosseous membrane.^16^ Injuries to these distal structures, which were not the focus of this study, may play a more significant role in rotational stiffness than the proximal soft tissue constraints alone.^13^^,^^15^ Second, the overall magnitude of rotational loss in our cohort was relatively small compared to the deficits seen in flexion and extension. It is possible that our sample size was insufficient to detect subtle associations between proximal soft tissue injuries and rotational limitations. Future larger-scale studies including evaluation of the interosseous membrane and distal radioulnar joint are warranted.

The finding that immobilization duration was not a significant predictor of outcomes must be interpreted with caution. The results of our power analysis indicate that this study lacked sufficient power to conclusively exclude a small, yet potentially clinically significant, effect of immobilization. However, the fact that immobilization remained nonsignificant even when analyzed as a continuous variable in a multivariate model strengthens our primary conclusion: the influence of the initial soft tissue injury appears to be substantially greater than that of the post-operative immobilization period in this cohort. This is a valuable outcome of this pilot investigation, as it quantifies the challenge of studying this variable and provides a clear, data-driven sample size target for a future, definitive trial.

These preliminary findings may have important clinical implications and suggest a potential for a paradigm shift in post-operative management. Current rehabilitation protocols are often standardized, with a primary focus on the timing of motion initiation. Our data suggest that a more nuanced, "soft tissue-directed" rehabilitation strategy, guided by pre-operative MRI findings, may be more effective. For instance, patients with an identified AC tear could be candidates for more aggressive, early, and protected extension-focused therapy to struggle capsular fibrosis. Conversely, those with pMCL tears might benefit from protocols that prioritize achieving and maintaining terminal flexion. This personalized approach warrants further investigation as it could lead to more efficient and effective rehabilitation.

This study has several important limitations. First, its retrospective design is susceptible to selection bias. Second, and most importantly, the small sample size limits the generalizability of the findings and rendered the study underpowered for the key comparison of immobilization durations. Third, as a single-center study, the results may reflect local practice patterns. Finally, while our regression model controlled for several key variables, other unmeasured confounders could exist. The consistent co-occurrence of aMCL and pMCL tears also prevented an independent analysis of the aMCL's contribution to stiffness.

The results of this pilot study provide a clear roadmap for future research. Based on compelling preliminary data and the calculated power analysis, a large-scale, multicenter, prospective cohort study is now warranted. Such a study should include over 250 patients to ensure adequate statistical power and should be designed to confirm the role of specific soft tissue injuries as independent predictors of stiffness, providing the high-level evidence needed to establish definitive, personalized post-operative management guidelines.

## Conclusion

In this pilot investigation of patients undergoing ORIF for displaced radial head fractures, post-operative stiffness was strongly and independently predicted by the presence of specific preoperative soft tissue injuries—AC and pMCL tears. The duration of post-operative immobilization was not a significant predictor of final ROM. These findings generate a compelling hypothesis that intrinsic soft tissue pathology is a more critical determinant of elbow stiffness than the duration of post-operative immobilization. This hypothesis now warrants rigorous investigation in a large, prospective, multicenter trial.

## Disclaimers:

Funding: No funding was disclosed by the authors.

Conflicts of interest: Each author certifies that he or she has no commercial associations (eg, consultancies, stock ownership, equity interest, patent/licensing arrangements, etc) that might pose a conflict of interest in connection with the submitted article.
